# Mineral Composition of Elements in Walnuts and Walnut Oils

**DOI:** 10.3390/ijerph15122674

**Published:** 2018-11-27

**Authors:** Iva Juranović Cindrić, Michaela Zeiner, Dora Hlebec

**Affiliations:** 1 Department of Chemistry, Faculty of Science, University of Zagreb, Horvatovac 102a, 10000 Zagreb, Croatia; ijuranovic@chem.pmf.hr (I.J.C.); dora.hlebec@gmail.com (D.H.); 2 Man-Environment-Technology Research Centre, School of Science and Technology, Örebro University, Gymnastikgatan 1, 70182 Örebro, Sweden; 3 Division of Analytical Chemistry, Department of Chemistry, BOKU—University of Natural Resources and Life Sciences, Muthgasse 18, 1190 Vienna, Austria

**Keywords:** essential elements, minerals, potentially toxic elements, PTWI, walnuts, walnut oil

## Abstract

Walnuts (*Juglans nigra*) are considered to be a functional food. In the present study, twenty one macro-, micro-, and trace elements (Al, As, B, Ba, Ca, Cd, Co, Cr, Cu, Fe, K, Li, Mg, Mn, Mo, Na, Ni, Pb, Se, Sr, and Zn) were selected to be determined in walnuts and walnut oils. The beneficial content of essential elements could be shown by the obtained results, the macro-elements Ca, K, Mg, and Na being present in nuts with 1062 mg/kg, 2771 mg/kg, 1426 mg/kg, and 42.3 mg/kg, respectively. Regarding micro- and trace elements, the following order (decreasing content) was found: Mn > Fe > Zn > B, Mo > Cu > Ni > Co > Al > Sr > Ba > Li > Pb > Se > Cr > As > Cd. Furthermore, the potentially toxic trace elements (As, Cd, Pb) determined were below the maximum allowed values in most of the investigated walnut samples. A comparison of oils and nuts revealed that the former contain lower concentrations of all elements analyzed except for Fe and Zn. This suggested the origin from contamination during oil processing. Fe influences the oil oxidation rate, thus its entry during production should be avoided.

## 1. Introduction

Walnuts are products that are consumed worldwide since they are highly regarded for their taste and as a source of important nutrients such as essential minerals, carbohydrates, phenolic compounds, vitamins, and polyunsaturated fatty acids [1,2,3,4]. Furthermore, they are a powerful source of vegetable proteins (14% dry matter) in vegetarian and vegan diet [5,6]. Bioactive compounds like phytic acid and pyrophosphate are also known to be minor components of walnuts, exhibiting the ability to be inhibitors of urolithiasis [7]. Thus, the incorporation of walnuts into the human diet is highly desirable, e.g., to achieve a positive effect on the heart, skin, and circulatory system. The prevention of coronary heart disease, diabetes, and cancer by the consumption of walnuts, including a consideration of the nutritional components in walnuts, has been recently studied [8,9,10,11,12]. Due to their nutritional and medicinal benefits, walnuts have even been considered to be a natural functional food. Eating walnuts may also be beneficial for hypertensive individuals, because they are rich in potassium, which eliminates sodium, which is highly implicated in the elevation of blood pressure. Copper, chromium, iron, and zinc are essential micronutrients for human health and important for human metabolism [13].

Whereas the organic compounds of walnuts are well-studied, there are only limited recent data on the mineral composition of walnuts. Ossai [14] reported the concentrations of Ca, Mg, K, Fe, and Na in walnuts to be higher than other elements. Applying enzymolysis to walnuts was used for studying the bioavailability of selected essential and harmful metals [15]. The solubility in the stomach (acidic environment) was found to be higher than in the intestinal tract (basic conditions). The quality of many foods such as their taste, appearance, texture, and stability depends on the content of minerals present. Furthermore, potentially toxic elements, which enter the fruits via the root uptake of the plant, must be considered when evaluating the nutritional value of walnuts. Knowledge of the mineral content of nuts is thus not only important for nutritional aspects, but also for toxicological reasons. It must be ensured that these metals are present below the respective PTWI (Provisional Tolerable Weekly Intake), PTDI (Provisional Tolerable daily Intake), or PTMI (Provisional Tolerable Monthly Intake) levels established by the FAO/WHO (2011) [16].

A great variety of digestion procedures for nuts have been reported, e.g., wet digestion using nitric acid [17], or a mixture of nitric acid and perchloric acid [14], but the procedures mostly rely on microwave-assisted digestion (MWD). Both types of MWD are used, i.e., open [18] and closed [19]. Advantages of MWD include simple control over the heating power and time heating, a reduced blank level, and little risk of contamination. Furthermore, these methods are often automated, quick, and allow for good reliability and efficiency.

The accurate analysis of food for nutritional content, contamination, the geographic source of the product, or authenticity is critical for regulatory and quality assurance. Several atomic spectroscopy techniques are available and selecting an appropriate one is the key to achieving accurate, reliable, real-world results. Atomic absorption spectrometry (AAS)—with flame (FAAS) [17,18] or electrothermal atomization (ETAAS) [14,20] was used as the analytical technique for element determination in walnuts or nut oil [21]. Using ETAAS, the sample is introduced directly into a graphite tube, which is then heated in a programmed series of steps to remove the solvent and major matrix components and to atomize the remaining sample. Furthermore, Inductively Coupled Plasma Optical Emission Spectroscopy (ICP-OES) provides very good detection capabilities, wide working ranges, and can be applied for element determination in walnuts after microwave digestion with nitric acid [19] or after ash treatment with hydrochloric acid [22]. Axial viewing provides better detection limits than those obtained via radial viewing by as much as a factor of 10. The most effective systems allow the plasma to be viewed in either orientation in a single analysis. With another simultaneous method, namely Inductively Coupled Plasma Mass Spectrometry (ICP-MS), even lower limits of detection for elements in nuts have been obtained [23,24,25,26].

The aim of the present investigation was to determine the mineral content of walnuts and walnut oils using acidic microwave-assisted digestion followed by ICP-AES or ICP-MS, as they are considered to contribute significantly to healthy nutrition. The study is focused on the following elements: As, B, Ba, Ca, Cd, Co, Cr, Cu, Fe, K, Li, Mg, Mn, Mo, Na, Ni, Pb, Se, Sr, and Zn. Comparing metal contents in oils and nuts leads to useful information for nutritional science.

## 2. Materials and Methods

### 2.1. Samples

Samples of shelled walnuts originating from Canada (*n* = 7) and France (*n* = 5) as well as walnut oils (*n* = 3) were purchased in duplicate from several grocery stores in Zagreb (Croatia) and Vienna (Austria) during the period 2014–2015. The smallest package size was acquired in each case.

### 2.2. Chemicals and Reagents

Supra pure nitric acid (HNO_3_) and hydrogen peroxide (H_2_O_2_) were purchased from Sigma (Munich, Germany). The multielement standard (ICP Multielement Standard IV) was purchased from Merck (Darmstadt, Germany). Ultra-pure water was prepared in an in-house instrument (resistivity > 18 MΩ). Strawberry leaves certified reference material (CRM: LGC7162) was obtained from LGC Standards (Middlesex, United Kingdom).

### 2.3. Sample Preparation

All the nut samples were shelled, dried at 105 °C for 24 h, and homogenized in a metal-free mortar prior to further sample preparation. Oil samples were used directly from the original bottles. Microwave-assisted digestion was performed using a MWS-2 Microwave System Speedwave Berghof (Berghof Laborprodukte GmbH, Eningen, Germany). Approximately 100 mg (nuts) or 500 mg (oils) weighed to the nearest 0.1 mg of the dried sample were put into a Teflon reaction vessel (in duplicate). Then, 5.00 mL HNO_3_ (50:50 *v*/*v*) and 1.00 mL H_2_O_2_ (1 mol/L) were added. The digestion consisted of the following three steps—15 min each: 1. P/W = 60%, T = 110 °C, 2. P/W = 75%, T = 170 °C and 3. P/W = 50%, T = 140 °C. The resulting clear solutions were made up to 10.0 mL with ultra-pure water. Blank solutions and CRM were treated with the same method of digestion.

### 2.4. Measurements

As, Cd, Li, Mo, and Se were quantified with ICP-SFMS (Element 2 from Thermo Fisher; Bremen, Germany). The instrument was equipped with a self-aspirating PFA microflow nebulizer (ESI: Elemental Scientific Inc., Omaha, NE, USA; flow of 100 µL/min), a PC3 cyclonic quartz chamber (ESI: Elemental Scientific Inc., Omaha, NE, USA; operated at 4 °C), a quartz injector pipe and torch, aluminum sampler and skimmer cone (all from Thermo Fisher, Bremen, Germany). The following instrumental conditions were applied: RF (radio frequency) power—1300 W; plasma gas flow—16 L/min; sample gas flow—1.06 L/min and auxiliary gas flow—0.86 L/min. The isotopes were analyzed at different resolutions, namely ^7^Li, ^82^Se, ^111^Cd at low resolution, ^98^Mo at medium resolution, and ^75^As at high resolution. The nominal mass resolutions were 350, 4500 and 10,000, respectively. At all resolution levels, ^115^In with 1.1 μg/L was used as the internal standard.

The other elements, i.e., Al, B, Ba, Ca, Co, Cr, Cu, Fe, K, Mg, Mn, Na, Ni, Pb, Sr, and Zn, were determined using a Prodigy High Dispersive ICP spectrometer (Teledyne Leeman, Hudson, NH, USA) working in simultaneous mode. The instrument was run with the following instrumental parameters: radiofrequency power of 1.1 kW; plasma gas flow rate (Ar) of 18 L/min; and auxillary gas flow rate (Ar) of 0.8 L/min.

The emission lines selected, presented in nm, are listed as the following: Al 396.152, B 249.677, Ba 455.403, Ca 315.887, Cr 267.716, Cu 224.700, Fe 259.940, K 766.491, Mg 280.271, Mn 259.372, Na 589.592, Ni 231.604, Pb 220.353, Sr 407.771, and Zn 213.856.

All the samples (digests of nuts, oils, CRM, and blank solutions) were measured in triplicate. The quantitative determinations were carried out using external calibration with multi-element standard solutions. The mean values of the blank solutions were subtracted from all the sample concentrations obtained.

### 2.5. Optimization and Characterization of the Analytical Method

Since no commercially available nut/seed reference material accredited for a wide range of inorganic constituents exists, other plant certified reference material (strawberry leaves) was used for the method validation (trueness). The overall repeatability of the instrument was determined by analyzing selected samples after calibration on different days. The precision expressed as relative standard deviation (RSD) was evaluated by measuring selected samples of each sample type five times. The sensitivity of the method was evaluated for each metal using the obtained slope of the calibration curve. The limits of detection (LOD; 3 σ) and limits of quantification (LOQ; 10 σ) were calculated according to Boumans [27].

### 2.6. Statistical Analysis

All analyses were carried out in triplicate. The mean and SD of each sample was calculated. The results of all brands did not differ at a statistically significant level (based on *p* < 0.05), and are thus presented together in Table 1.

## 3. Results and Discussion

### 3.1. Analytical Method

The figures of merit obtained for the analytical methods used in the investigation presented are in the range for the determination of trace-, micro-, and macro-elements in biological samples. The limits of detection (LOD) for the digested nuts and oils were below 2 μg/g for all analytes. The recoveries determined with the standard reference material of strawberry leaves ranged from 82% to 116% for the ICP-OES, and from 84% to 112% for the certified elements determined using ICP-MS. The sensitivity of the method was evaluated by the slopes of the calibration curves for all elements, with coefficients of determination (R^2^) higher than 0.9995 in all cases. The precision ranged from 0.1% to 2.3%, and the within-day repeatability ranged from 0.9 to 3.3%. The overall uncertainty of measurement was estimated to be <6% (coverage factor k = 2) for all analytes and sample types.

### 3.2. Elemental Content of Walnuts

The elemental composition in the walnut samples analyzed is presented in Table 1 alongside the literature data. In the present investigation, more elements were determined in walnuts than in the majority of papers published to date.

Considering macro essential elements important for humans, the walnuts analyzed contained Ca, K, Mg, and Na in mg/kg: 1062, 2771, 1426, and 42.3, respectively. Apart from the study by Aryapak et al. [17], who found about twice as much Ca, the literature data are similar to the obtained results [26,28]. Lower concentrations of Ca were reported by Comulescu et al. [23], Ossai [14], and Laverdine et al. [18] and by Çağlarιrmak [22]. Conversely, higher concentration ranges were reported for K by other working groups [14,17,18,19,23,26]. Regarding Na, the contents in walnuts vary over a wide range, from smaller [23] to higher values [17] than those found in the present study, and there were also similar values to our data [18,19].

The values obtained for Mg are in good agreement with those reported by Özcan [19], Laverdine et al. [18] and Ossai [14], whereas much higher results are given by Aryapak et al. [17] and Rodushkin et al. [26].

The results indicate that walnuts are a useful contributor to the supply of essential elements to the human body. Differences compared to literature data can be explained by the influence of cultivar, year of harvest, and growing site.

Although certain essential elements are only required in trace amounts, their deficiencies in the human population are widespread; not even 50% of the US populations covers its Mg needs via nutrition [29]. Some important micro- and trace elements (essential and non-essential) for many metabolic and physiological functions, like B, Ba, Cd, Co, Cr, Cu, Fe, Li, Mn, Mo Ni, Pb, Se, Sr, and Zn, are present in walnuts at µg/kg to mg/kg level and their contents are given in Table 1. In general, the order of the concentrations of the elements in the nut samples is Mn > Fe > Zn >B, Mo > Cu > Ni > Co > Al > Sr > Ba > Li > Pb > Se > Cr > As > Cd. In contrast to the major elements, the concentration ranges of minor and trace elements differ much more. Regarding Cd and Cr, the values vary even by a factor of two or more orders of magnitude. Whereas the major elements are mainly determined by the composition of the mother rock, where the nut trees are grown, the soil contents of trace elements are influenced by environmental factors, such as dry and wet deposition. Thus, even in remote areas, elevated levels of pollutants may be found.

Since potentially toxic metals (e.g., Pb, As, and Cd) may enter the food chain as a result of their uptake by walnuts, it is necessary to assess the levels of toxic metal and to report possible contamination that would represent a health hazard. The FAO/WHO [16] has set a limit for heavy metal intakes based on body weight (see Table 2). As can be seen from Table 2, no harmful impact from these elements by the consumption of up to 100 g nuts per day can be inferred.

Lead is one of the representative metals whose concentration represents a reliable index of environmental pollution. In walnut samples, Pb is found in the range of 0.331–0.527 mg/kg, slightly above the standard limit in China [29], which for nuts is 0.2 mg/kg. A similar content of Pb in walnuts is reported by Cabrera et al. [20].

Arsenic can cause gastrointestinal tract injuries and cardiac disorders. The As levels in walnut samples up to 0.06 mg/kg are considerably lower than the limit set by the FDA [30] for As in fruit and vegetables (1.4 mg/kg).

Cadmium content in walnuts was found to be 0.0039 mg/kg. The limit stated by the FAO/WHO [16] is a maximum monthly uptake of 0.025 mg/kg bw. The values obtained for Cd vary from those reported in literature (Table 1). This tendency is often observed for ultra-trace elements, resulting from anthropological impact. 

Regarding the stated limits for provisional tolerable intake, the nuts analyzed can be considered as safe food for humans.

### 3.3. Elemental Content of Walnut Oils

The quality of walnut oils as well as other edible oils is directly related to the content of trace metals. Especially important are trace elements like Fe, Cu, Ca, Mg, Co, Ni, and Mn, which are known to increase the rate of oil oxidation, especially Cu and Ni, which affect quality by promoting the oxidative degradation of oils [31]. Other elements such as Cr, Cd, and Pb are important for their toxicity and metabolic roles. As can be seen from Table 3, the metal and metalloid contents found in walnut oils are in general lower than those in the nuts, indicating that the elements analyzed are mainly bound to the proteins of the walnut kernels; thus by pressing they remain in the residue. However, higher Fe and Zn contents were found in the oil than in the nut, which may be due to contamination during oil processing. Since Fe is one of the elements that increases the oil oxidation rate, its entry during production should be kept as low as possible.

In the literature, there are many studies on the determination of trace elements in edible oils and their controls for human health using different sample preparation procedures, pointing out the difficulties that arise due to the characteristics of the oil matrix [21,32].

Even though walnut oils are considered as very healthy and a useful supplement for the human diet, their inorganic composition is not well-documented. Thus, oils originating from almonds or hazelnuts are used for discussion. Compared to the literature data, the Cd content found is lower in other types of edible oil, such as hazelnut oil [21,33,34]. Conversely, the results for Cr are higher than in all reported hazelnut oils [32,33,34]. Regarding Cu, lower contents are given by other working groups [21,33,34] except for Juranović Cindrić et al. [32]. Apart from the values reported by Mendil et al. [21], the Fe content in all other hazelnut oils was lower by a factor of two [32] or even one hundred [33]. The Zn content determined in walnut oil was found to be 38.6 mg/kg, which is considerably higher than in hazelnut oil [21,33,34]. Data for only four elements, namely Mn, Ni, Pb, and Sr, are given by Savio et al. [35] for almond oil, according to which the latter three are below LOD.

## 4. Conclusions

In this investigation, more elements contributing to the mineral composition were determined in walnuts than in the majority of papers published to date. The results indicate that walnuts are a useful contributor to the supply of essential elements to the human body. Regarding the stated limits for provisional tolerable intake, the nuts analyzed can be considered as safe for consumption. The differences compared to literature data can be explained by the influence of cultivar, the year of harvest, and the growing site. The data presented refer to nuts originating from Canada and France, two main contributors to the world’s walnut market. Thus, they are of limited use for specific risk assessment studies of other areas.

The metals present are mainly bound to the proteins of the walnut kernel; thus by pressing them to obtain oil, they remain in the residue. Only a small quantity remains in the oil obtained. However, higher Fe, Sr, and Zn contents were found in the oil than in the nut, which may be due to contamination during oil processing.

## Figures and Tables

**Table 1:** The content of elements in walnuts as compared to literature values (mg/kg).

Analyte	Mean	Min	Max	Rodushkin et al. [26]	Özcan [18]	Lavedrine et al. [19]	Arpadjan-Ganeva et al. [15]	Moreda–Piñeiro et al. [25]	Cabrera et al. [20]	Cosmulescu et al. [23]	Ossai [14]	Scherz & Kirchhoff [13]	Yin et al. [24]	Aryapak & Ziarati [17]
Al	5.05	2.93	6.533	0.34 ± 0.23	5.8 ± 1.0			<1.5–69	2.7–5.4	0.02–5.25				
As	0.027	<LOD	0.057	0.007 ± 0.006									0.12 ± 0.04	
B	13.3	6.83	22.5	15 ± 2	58.8 ± 16.2									
Ba	2.32	1.44	4.54	3.1 ± 2.8	5.9 ± 2.4			<0.026–13787					0.45 ± 0.06	
Ca	1062	866	1435	1200 ± 300	1108.6 ± 13.7	580–910				317–908	910 ± 50			2560 ± 176
Cd	0.0039	0.0018	0.0066	0.00065 ± 0.00069		11–14	0.009–0.02		<LOD–0.009				0.02	
Co	5.50	2.35	8.21	0.045 ± 0.035	0.24 ± 0.008				0.30–0.38				0.02 ± 0.001	
Cr	0.028	0.017	0.037	0.0013 ± 0.0007	8.9 ± 0.7		0.04–0.18			2.55–6.92	<LOD		<LOD	
Cu	8.38	5.12	20.8	16 ± 3	3.8 ± 2.2		15–17	109–3817	20–26	14.1–32.2			8.8 ± 0.15	281 ± 307
Fe	27.4	13.6	39.1	41 ± 11	32.4 ± 4.3	18–29	22–30	4.9–22	20–24	38.2–59.3	30 ± 2			5017 ± 73
K	2771	2006	3221	5100 ± 1000	4627.6 ± 34.7	3720–4870		4800–13700	12–56	3571–4996	3110 ± 40			5819 ± 277
Li	0.632	<LOD	1.81	5.6 ± 1.9	0.68 ± 0.06								0.01 ± 0.01	
Mg	1426	875	1824	4500 ± 800	1089.9± 26.3	1290–2020		1000–5540		1892–2781	1164 ± 56			6363 ± 186
Mn	31.7	13.7	46.0	34 ± 15	46.3 ± 6.5	11–43	39–42	7.7–84		31.3–176	<LOD		10 ± 0.1	
Mo	13.3	5.06	21.7	0.48 ± 0.34									0.21	
Na	42.3	18.7	94.3	1.8 ± 0.8	44.7 ± 6.5	3–67				1.34–23.9	72 ± 3			625 ± 197
Ni	6.05	2.14	9.90	1.6 ± 1.5	2.4 ± 0.6			<0.1–13	0.18–0.20					
Pb	0.331	0.143	0.527	0.00025 ± 0.00012					0.20–0.26		<LOD		<LOD	
Se	0.194	0.077	0.301	0.012 ± 0.01	15.7 ± 2.9					0.01–0.05	<LOD		<LOD	0.03 ± 0.015
Sr	4.41	2.62	9.16	6.5 ± 3.2	2.4 ± 0.6			0.15–198		1.6–5.4			3.4 ± 0.01	
Zn	24.0	20.3	32.8	33 ± 8	26.4 ± 3.2	12–19	27–37	12–63	26–40	19.5–36.1	19 ± 2	25–37	20 ± 1.1	1380 ± 452

**Table 2:** Provisional tolerable intakes for selected metals and estimated intake for consumption of 100 g nuts (considering a water content of 3%).

Element	PTI *		Uptake	Mass in 100 g Nuts
	(mg/kg bw)	Per	(mg/60 kg Person)	(mg)
Al	1.0	week	60	0.49
As	0.003	day	0.180	0.0026
Cd	0.025	month	1.500	0.00038
Pb	withdrawn in 2010			0.032

* FAO/WHO: Food Standards Programme Codex Committee on Contaminants in Foods. Fifth Session. The Hague, The Netherlands, 2011 [16].

**Table 3:** The content of elements in walnut oils as compared to literature values for similar oil types (mg/kg).

Analyte	Mean	Min.	Max.	Mendil et al. [21] Hazelnut Oil	Juranović Cindrić et al. [32] Hazelnut Oil	Bakircioglu et al. [33] Hazelnut Oil	Gunduz et al. [34] Hazelnut Oil	Savio et al. [35] Almond Oil
Al	<LOD	<LOD	<LOD		0.27			
As	0.021	<LOD	0.046					
B	<LOD	<LOD	<LOD					
Ba	<LOD	<LOD	<LOD					
Cd	0.0012	<LOD	0.0032	4.57 ± 0.4		0.010–0.051	0.0142–0.0196	
Co	0.0018	0.0009	0.0029	0.54 ± 0.04	0.096			
Cr	0.064	0.002	0.152		<0.001	0.008–0.852	<LOD	
Cu	0.156	0.073	0.529	0.05 ± 0.004	0.5	0.030–4.504	0.0182–0.0229	
Fe	31.8	5.35	86.4	127.0 ± 11.4	15.5	0.222–12.588		
Li	0.009	<LOD	0.027					
Mn	0.041	0.012	0.099	0.13 ± 0.01	0.44	0.026–0.054		0.009 ± 0.002
Mo	0.0026	0.0018	0.0034					<LOD
Ni	0.035	0.0070	0.069			0.478–2.182	0.0096–0.0132	<LOD
Pb	0.147	0.077	0.335	0.01 ± 0.01	<0.001	0.021–0.114	0.0142	
Se	0.0061	<LOD	0.0073					
Sr	0.041	<LOD	0.081					<LOD
Zn	38.6	9.7	85.0	1.15 ± 0.1	3.4	1.136–8.982		
